# Robot-assisted vs. laparoscopic right hemicolectomy in octogenarians and nonagenarians: an analysis of the US nationwide inpatient sample 2005–2018

**DOI:** 10.1007/s40520-024-02833-4

**Published:** 2024-09-23

**Authors:** Chien-Chang Lu, Chi-Tung Lu, Kai-Yen Chang, Wang Chun-Li, Chien-Ying Wu

**Affiliations:** 1 Department of Colorectal Surgery, Kaohsiung Chang Gung Memorial Hospital, Chang Gung University College of Medicine, No. 123, Dapi Road, Niaosong District, Kaohsiung City, Taiwan; 2 Kaohsiung Chang Gung Memorial Hospital, Kaohsiung, Taiwan

**Keywords:** Colorectal cancer (CRC), Laparoscopic right Hemicolectomy (LRH), Nationwide Inpatient Sample (NIS), Nonagenarians, Octogenarians, Robot-assisted right Hemicolectomy (RARH)

## Abstract

**Background:**

Colorectal cancer (CRC) is a significant health concern, particularly among older adults. Outcomes between laparoscopic and robot-assisted surgeries for right-sided colon cancers in the oldest old population have yet to be evaluated despite increased use of these surgeries.

**Aim:**

This study aimed to compare clinical outcomes after robot-assisted right hemicolectomy (RARH) versus laparoscopic right hemicolectomy (LRH) in octogenarian and nonagenarian patients.

**Methods:**

This population-based, retrospective and observational study analyzed the data of adults ≥ 80 years old diagnosed with right-side colon cancer who received RARH or LRH. All data were extracted from the US National Inpatient Sample (NIS) database 2005–2018. Associations between type of surgery and in-hospital outcomes were determined using univariate and multivariable logistic regression and linear regression analysis.

**Results:**

Data of 7,550 patients (representing 37,126 hospitalized patients in the U.S.) were analyzed. Mean age of the study population was 84.8 years, 61.4% were females, and 79.1% were non-smokers. After adjusting for relevant confounders, regression analysis showed that patients undergoing RARH had a significantly shorter LOS (adjusted Beta (aBeta), -0.24, 95% CI: -0.32, -0.15) but greater total hospital costs (aBeta, 26.54, 95% CI: 24.64, 28.44) than patients undergoing LRH. No significant differences in mortality, perioperative complications, and risk of unfavorable discharge were observed between the two procedures (*p* > 0.05). Stratified analyses by frailty status revealed consistent results.

**Conclusions:**

RARH is associated with a significantly shorter LOS but higher total hospital costs than LRH among octogenarians and nonagenarians. Other short-term outcomes for this population are similar between the two procedures, including in-hospital mortality, perioperative complications, and unfavorable discharge. These findings also apply to frail patients.

**Supplementary Information:**

The online version contains supplementary material available at 10.1007/s40520-024-02833-4.

## Introduction

Colorectal cancer (CRC) is the third most commonly diagnosed cancer and the third most common cause of cancer-related death in both men and women in the United States (US) [1]. CRC incidence and mortality increase significantly with age, with the median age of diagnosis in developed nations approaching 70 years. Male sex, excessive alcohol use, high consumption of red and processed meat, smoking, lack of physical inactivity, having a family history of CRC, and being overweight or obese are recognized risk factors independent of advanced age [2].

Among colon cancers, specifically, right-sided colon cancer refers to the primary tumors that originate at the cecum, ascending colon, and the first two-thirds of the transverse colon, while left-sided colon cancer refers to tumors in the remaining third of the transverse colon, descending colon, sigmoid colon, and rectum [3]. These two types of colon cancer have distinct characteristics in etiology, histological features, disease progression, and survival outcomes [4]. Typically, right-sided colon cancer is more likely to present with a larger tumor size, higher tumor grade or lack of differentiation, and high immunogenicity [5]. It also tends to metastasize to the peritoneal region [6] and is more frequently diagnosed in older adults [7], particularly females [8], compared to left-sided tumors. Although the 5-year mortality for right-sided colon cancer is similar to that of left-sided CRC [8, 9], the prognosis for advanced right-sided cancer is significantly worse, with higher recurrence rates after surgical resection than left-sided tumors [9, 10].

Surgical resection is considered the most effective approach for treating CRC and achieving long-term survival [11, 12]. Minimally invasive surgery has been increasingly employed considering its advantages, especially smaller incisions, shorter recovery time, and reduced postoperative complications [11, 13]. Due to greater degrees of rotation, articulation, and tri-dimensional imaging, robotic-assisted right hemicolectomy (RARH) may have technological advantages over the conventional laparoscopic right colectomy (LRH). Studies comparing outcomes between the two procedures have reported that RARH had a longer operative duration, lower expected blood loss, a lower rate of overall complications, and greater total expenditures when compared to LRH, while other results were comparable [14].

The octogenarian and nonagenarian population represents a rapidly growing age group in the US, with a higher likelihood of requiring surgical intervention for age-related conditions such as CRC. Notably, older adults with right-sided cancers are shown to be less likely to receive standard treatments, which may contribute to poorer outcomes [15]. While LRH and RARH have been performed extensively within the general population, their specific outcomes in octogenarians and nonagenarians remain poorly investigated.

Therefore, the objective of this study was to assess and compare the clinical outcomes of LRH and RARH in octogenarian and nonagenarian patients using data from the US Nationwide Inpatient Sample (NIS) database.

## Materials and methods

### Study design and data source

This population-based, retrospective, observational study extracted all data from the United States (US) National Inpatient Sample (NIS) database, which is the largest fully paid continuum of hospital care data in the US. NIS contains data of approximately 8 million hospitalizations per year. The Health Care Cost and Utilization Project (HCUP) of the National Institutes of Health (NIH) manages the NIS database. NIS has data on primary and secondary diagnoses, primary and secondary procedures, length of stay (LOS), patient demographics, admission and discharge status, expected source of payment, and hospital characteristics. Hospital characteristics include bed size, location, teaching status, and hospital geographic area. The NIS database is updated annually and draws patient data from approximately 1,050 hospitals in 44 US states, representing a stratified sample of 20% of US community hospitals as defined by the American Hospital Association [16].

### Ethics statement

This study complies with the data use agreement between NIS and the Healthcare Cost and Utilization Project (HCUP). All data were obtained by request to the Online HCUP Central Distributor who manages the database. Because this study analyzed secondary data from the NIS database, patients and the public were not directly involved. The study protocol was submitted to the Institutional Review Board (IRB) of HCUP-339M32DWW, which waived IRB approval for the study. Since all data in the NIS database is de-identified and patients remain anonymous, the requirement for informed consent was also waived.

### Study population

This study included adults aged ≥ 80 years with right-side colon cancer who received robot-assisted right hemicolectomy (RARH) or Laparoscopic right hemicolectomy (LRH). The International Classification of Diseases, Ninth Revision (ICD-9) and Tenth Revision (ICD-10) diagnostic codes were used to identify eligible patients in the NIS database admitted to US hospitals between 2005 and 2018. Patients with metastatic disease and/or missing information on sex and main endpoints, such as mortality, length of stay (LOS), or hospital costs, were excluded.

### Main outcomes and study variables

Primary outcomes included in-hospital mortality, length of stay (LOS), unfavorable discharge (to long-term care or nursing homes), hospital costs, and postoperative complications. Major postoperative complications included death, acute myocardial infarction (AMI), venous thromboembolism (VTE), pneumonia, sepsis, infection, major blood loss, respiratory failure, mechanical ventilation, acute kidney injury (AKI), postoperative ileus, abdominal abscess/ fistula, and wound disruption.

### Covariates

Patients’ characteristics evaluated included age, sex, race, household income level, insurance status (primary payer), admission type (elective or emergent), weekend admission, year of admission, smoking status and frailty status. Hospital-related characteristics (bed number, location/teaching status, hospital region) were extracted from the database as part of the comprehensive data available for all patients. Major comorbidities, including ischemic heart disease, congestive heart failure, diabetes, chronic obstructive pulmonary disease (COPD), cerebrovascular disease, obesity, severe liver disease, chronic kidney disease (CKD), systemic connective tissue disorders, and coagulopathy were identified using ICD-9 and ICD-10 diagnostic codes (Supplemental Table S1). With respect to frailty status, we used the Hospital Frailty Risk Score (HFRS)_ developed and validated by Gilbert et al. [17]. This score uses ICD codes to assign weights to various conditions, such as heart failure, chronic pulmonary disease, and volume depletion. The score is calculated by summing points assigned to each ICD code, with higher scores indicating greater frailty risk. Designed for screening frailty in hospitalized elderly patients aged 75 and above, it is regarded as a cost-effective tool that integrates into hospital information systems and performs comparably to other frailty and risk assessment tools. In this study, frailty is categorized into two risk levels: not frail (HFRS < 5) and frail (HFRS ≥ 5), consistent with previous studies [18, 19].

### Statistical analysis

Since the NIS database covers 20% samples of the USA annual inpatient admissions, weighted samples (TRENDWT before 2011; DISCWT after 2012), stratum (NIS_STRATUM), clusters (HOSPID) were used to generate national estimates for all analyses. SAS analyzes sample survey data using the SURVEY procedure. Descriptive statistics of patients with right-side colon cancer who underwent robot-assisted right hemicolectomy (RARH) or laparoscopic right hemicolectomy (LRH) are presented as numbers (n) and weighted percentages (%) or mean and standard error (SE). Categorical data were analyzed by PROC SURVEYFREQ statement, and continuous data were analyzed by PROC SURVEYREG statement. Odds ratios (ORs) and 95% confidence intervals (CIs) were calculated for dichotomized outcomes using logistic regression analysis and Beta using linear regression analysis. Variables with significant differences between the two comparison groups were entered into multivariable regression models after adjustments for possible confounders. All p-values are two-sided, and *p* < 0.05 was considered statistically significant. All statistical analyses were performed using the statistical software package SAS software version 9.4 (SAS Institute Inc., Cary, NC, USA).

## Results

### Study population selection

**Fig. 1:**
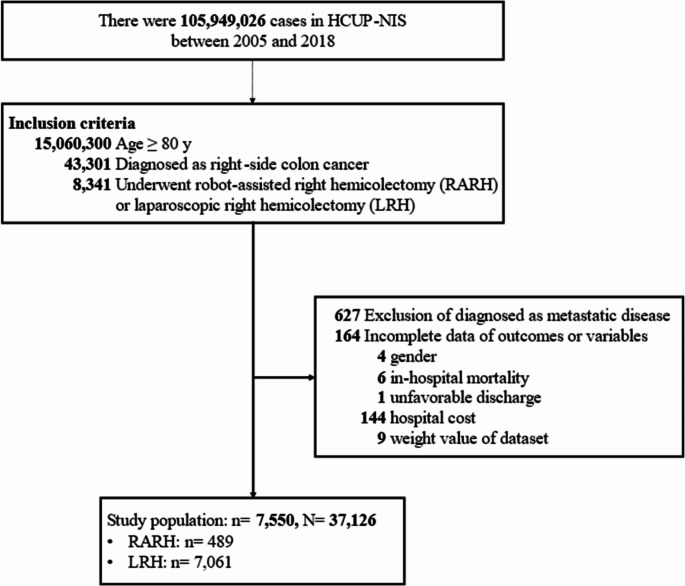
Flow diagram of patient selection

The flow diagram of study population selection is summarized in Fig. 1. The present study identified a total of 8,341 patients with right-side colon cancer undergoing RARH or LRH in the NIS database between 2005 and 2018. Patients with metastatic disease (*n* = 627) or those who had missing information on study outcomes or variables (*n* = 164) were excluded. Finally, 7,550 patients were enrolled as the study cohort (representing 37,126 hospitalized patients in the entire U.S.). Among these, 489 (6.5%) patients underwent RARH and 7,061 (93.5%) underwent LRH. (Fig. 1).

### Characteristics of the study population

Patients’ baseline characteristics are summarized in Table 1. The mean age of the study population was 84.8 ± 0.04 years, 61.4% were females, and 79.1% were non-smokers. Patients undergoing RARH had higher percentages of smokers, without frailty, insurance covered by Medicare or Medicaid, were admitted emergently at non-weekend, and were admitted to urban-teaching hospitals than those undergoing LRH (all *p* < 0.05).

**Table 1:** Characteristics of patients with right-side colon cancer undergoing RARH or LRH

Characteristics	Total		RARH		LRH		p-value		
	(*n* = 7550)		(*n* = 489)		(*n* = 7061)				
**Age**,** mean years (SD)**	84.8 ± 0.04		84.3 ± 0.11		84.8 ± 0.04		**< 0.001**		
80–89	6559 (86.9)		439 (89.7)		6120 (86.7)		**0.039**		
90+	991 (13.1)		50 (10.3)		941 (13.3)				
**Sex**							0.738		
Male	2913 (38.6)		185 (37.9)		2728 (38.6)				
Female	4637 (61.4)		304 (62.1)		4333 (61.4)				
**Insurance status**							**0.041**		
Medicare/Medicaid	7139 (94.6)		465 (95.2)		6674 (94.6)				
Private including HMO	354 (4.6)		16 (3.3)		338 (4.7)				
Self-pay/no-charge/other	53 (0.7)		7 (1.5)		46 (0.7)				
Missing	4		1		3				
**Household income**							0.882		
Quartile1	1453 (19.5)		95 (19.6)		1358 (19.5)				
Quartile2	1943 (26.1)		131 (27.1)		1812 (26.0)				
Quartile3	1976 (26.5)		130 (26.8)		1846 (26.5)				
Quartile4	2092 (27.9)		128 (26.5)		1964 (28.0)				
Missing	86		5		81				
**Smoking**							**< 0.001**		
No	5982 (79.1)		358 (73.2)		5624 (79.5)				
Yes	1568 (20.9)		131 (26.8)		1437 (20.5)				
**Emergent admission**							**< 0.001**		
No	2170 (28.9)		91 (18.6)		2079 (29.6)				
Yes	5360 (71.1)		397 (81.4)		4963 (70.4)				
Missing	20		1		19				
**Weekend admission**							**0.018**		
No	7076 (93.7)		470 (96.1)		6606 (93.6)				
Yes	474 (6.3)		19 (3.9)		455 (6.4)				
Missing	0		0		0				
**Admission year**							**< 0.001**		
2005–2009	1288 (16.8)		8 (1.5)		1280 (17.8)				
2010–2014	3784 (49.9)		168 (34.2)		3616 (51.0)				
2015–2018	2478 (33.4)		313 (64.3)		2165 (31.2)				
**Hospital bed size**							0.332		
Small	1020 (13.4)		74 (15.2)		946 (13.2)				
Medium	2064 (27.5)		123 (25.3)		1941 (27.7)				
Large	4443 (59.1)		292 (59.5)		4151 (59.1)				
Missing	23		0		23				
**Hospital location/teaching status**							**< 0.001**		
Rural	584 (7.7)		20 (4.1)		564 (8.0)				
Urban nonteaching	2666 (35.4)		160 (32.5)		2506 (35.6)				
Urban teaching	4277 (56.9)		309 (63.4)		3968 (56.4)				
Missing	23		0		23				
**CCI**							0.935		
0–1	5145 (68.1)		331 (67.7)		4814 (68.1)				
2–3	1792 (23.8)		116 (23.8)		1676 (23.8)				
4+	613 (8.2)		42 (8.6)		571 (8.1)				
**Frailty status**							**< 0.001**		
Not frail	3670 (48.7)		292 (59.8)		3378 (48.0)				
Frail	3880 (51.3)		197 (40.2)		3683 (52.0)				
**Major comorbidity**									
CKD	1058 (14.1)		77 (15.8)		981 (14.0)		0.248		
Ischemic heart disease	2221 (29.4)		128 (26.2)		2093 (29.7)		0.094		
Congestive heart failure	1131 (15.0)		59 (12.0)		1072 (15.2)		**0.049**		
COPD	1191 (15.8)		80 (16.4)		1111 (15.7)		0.702		
Cerebrovascular disease	297 (3.9)		18 (3.7)		279 (3.9)		0.737		
Systemic connective tissue disorders	225 (3.0)		17 (3.5)		208 (3.0)		0.505		
Coagulopathy	237 (3.1)		14 (2.9)		223 (3.2)		0.720		
DM	1712 (22.7)		119 (24.3)		1593 (22.5)		0.324		
Obesity	538 (7.2)		42 (8.6)		496 (7.1)		0.198		

Continuous variables are presented as mean ± SE; categorical variables are presented as unweighted counts (weighted percentage)

P-values < 0.05 are shown in bold

*Abbreviations* RARH, robot-assisted right hemicolectomy; LRH, laparoscopic right hemicolectomy; HMO, Health maintenance organization; CCI, Charlson Comorbidity Index; CKD, chronic kidney disease; COPD, chronic obstructive pulmonary disease; DM, diabetes mellitus

### In-hospital outcomes

In-hospital outcomes are summarized in Table 2. The percentages of in-hospital mortality, presence of any complication and unfavorable discharge in the total study population were 1.7%, 53.0% and 28.5%, respectively. Mean hospital LOS was7.6 ± 0.1 days and the mean total hospital costs were 75.6 ± 0.8 thousand US dollars. Lower percentages of infection, major blood loss and postoperative ileus, and shorter mean hospital LOS were observed in patients undergoing RARH compared to those undergoing LRH (all *p* < 0.05). Higher mean total hospital costs were observed in the RARH group than in the LRH group (*p* < 0.05).

**Table 2:** In-hospital outcomes in patients with right-side colon cancer undergoing RARH or LRH

		Total		RARH		LRH		p-value	
		(*n* = 7550)		(*n* = 489)		(*n* = 7061)			
**In-hospital mortality**		125 (1.7)		9 (1.8)		116 (1.6)		0.683	
**Postoperative complication**,** any**		4007 (53.0)		221 (45.2)		3786 (53.6)		**< 0.001**	
AMI		90 (1.2)		2 (0.4)		88 (1.2)		0.104	
VTE		218 (2.9)		16 (3.3)		202 (2.9)		0.562	
Pneumonia		235 (3.1)		11 (2.3)		224 (3.2)		0.198	
Sepsis		340 (4.5)		19 (3.9)		321 (4.5)		0.475	
Infection		803 (10.6)		38 (7.7)		765 (10.8)		**0.032**	
Major blood loss		2624 (34.8)		144 (29.5)		2480 (35.1)		**0.013**	
Respiratory failure/ mechanical ventilation		488 (6.4)		31 (6.3)		457 (6.4)		0.892	
Acute kidney injury		687 (9.1)		48 (9.8)		639 (9.1)		0.572	
Postoperative ileus		1407 (18.5)		58 (11.9)		1349 (19.0)		**< 0.001**	
Abdominal abscess/ fistula		111 (1.5)		5 (1.0)		106 (1.5)		0.356	
Wound disruption		37 (0.5)		5 (1.0)		32 (0.5)		0.057	
**LOS**,** days**^**a**^		7.6 ± 0.1		6.4 ± 0.2		7.7 ± 0.1		**< 0.001**	
**Unfavorable discharge** ^**a**^		2118 (28.5)		126 (26.2)		1992 (28.7)		0.416	
**Hospital costs (per thousand dollars)**		75.6 ± 0.8		102.0 ± 2.7		73.8 ± 0.8		**< 0.001**	

Continuous variables are presented as mean ± SE; categorical variables are presented as unweighted counts (weighted percentage)

^a^ Patients with in-hospital mortality were excluded

P-value < 0.05 are shown in bold

*Abbreviations* RARH, robot-assisted right hemicolectomy; LRH, laparoscopic right hemicolectomy; AMI, acute myocardial infarction; VTE, *venous thromboembolism*; LOS, length of stay in hospital

### Associations between type of surgery and in-hospital outcomes

Table 3 shows the associations between type of surgery and in-hospital outcomes. After adjustments, patients undergoing RARH had a significantly shorter hospital LOS (adjusted Beta (aBeta), -0.24, 95% CI: -0.32, -0.15) and higher hospital costs (aBeta, 26.54, 95% CI: 24.64, 28.44) than patients undergoing LRH. In contrast, no significant differences were observed between the two types of surgery on in-hospital mortality, unfavorable discharge, and complications.

**Table 3:** Associations between types of minimally-invasive surgery and in-hospital outcomes

	Univariate		Multivariable	
	OR/Beta (95%CI)	*p*-value	aOR/aBeta (95%CI)	*p*-value
**In-hospital mortality** ^**a**^				
RARH	1.13 (0.64, 2.00)	0.684	1.71 (0.94, 3.10)	0.080
LRH	Ref.		Ref.	
**Complication (any)** ^**b**^				
RARH	**0.72 (0.60**,** 0.86)**	**< 0.001**	1.04 (0.85, 1.27)	0.713
LRH	Ref.		Ref.	
**LOS**,** days**^**c, f**^				
RARH	**-1.24 (-1.43**,** -1.05)**	**< 0.001**	**-0.24 (-0.32**,** -0.15)**	**< 0.001**
LRH	Ref.		Ref.	
**Unfavorable discharge** ^**d, f**^				
RARH	0.88 (0.72, 1.09)	0.236	1.10 (0.89, 1.37)	0.368
LRH	Ref.		Ref.	
**Hospital costs (per thousand dollars)** ^**e**^				
RARH	**28.26 (18.21**,** 38.32)**	**< 0.001**	**26.54 (24.64**,** 28.44)**	**< 0.001**
LRH	Ref.		Ref.	

Abbreviations: RARH, robot-assisted right hemicolectomy; LRH, laparoscopic right hemicolectomy; CCI, Charlson Comorbidity Index; OR, odds ratio; aOR, adjusted odds ratio; aBeta, adjusted Beta; CI, confidence interval; LOS, length of stay

^a^ Adjusted for sex, insurance status, smoking, emergent admission, weekend admission, Hospital bed size, hospital location/teaching status, CCI and frailty status

^b^ Adjusted for age group, household income, smoking, emergent admission, weekend admission, admission year, hospital location/teaching status, CCI and frailty status

^c^ Adjusted for age group, household income, smoking, emergent admission, weekend admission, admission year, Hospital bed size, hospital location/teaching status and frailty status

^d^ Adjusted for age group, sex, smoking, emergent admission, weekend admission, admission year, CCI and frailty status

^e^ Adjusted for age group, sex, household income, emergent admission, weekend admission, admission year, hospital location/teaching status, CCI and frailty status

^f^ Excluded patients with in-hospital mortality

P-values < 0.05 are shown in bold

### Stratified associations between obesity status and outcomes in patients with HF undergoing AF catheter ablation

Table 4 shows the stratified associations between type of surgery and in-hospital outcomes by frailty status. In frail subgroup, patients undergoing RARH had a significantly lower LOS (aBeta, -0.22, 95% CI: -0.29, -0.15) and higher total hospital costs (aBeta, 25.04, 95% CI: 24.11, 25.98) than LRH. Similar results were observed in the subgroup without frailty (LOS [aBeta, -0.32, 95% CI: -0.43, -0.22]; hospital costs [aBeta, 28.98, 95% CI: 21.02, 36.94], respectively).

**Table 4:** Associations between types of minimally-invasive surgery and in-hospital outcomes of surgery and in-hospital outcomes, stratified by frailty status

Subgroup	Type of surgery	Multivariable	
		aOR/aBeta (95% CI)	*p*-value
Not frail			
In-hospital mortality ^a^	RARH vs. LRH	1.79 (0.58, 5.54)	0.314
Complication (any) ^b^	RARH vs. LRH	1.27 (0.99, 1.64)	0.064
LOS ^c, f^	RARH vs. LRH	-0.22 (-0.29, -0.15)	**< 0.001**
Unfavorable discharge ^d, f^	RARH vs. LRH	0.98 (0.70, 1.37)	0.902
Hospital cost (per thousand dollars) ^e^	RARH vs. LRH	25.04 (24.11, 25.98)	**< 0.001**
Frail			
In-hospital mortality ^a^	RARH vs. LRH	1.68 (0.83, 3.40)	0.147
Complication (any) ^b^	RARH vs. LRH	0.79 (0.59, 1.06)	0.112
LOS ^c^	RARH vs. LRH	-0.32 (-0.43, -0.22)	**< 0.001**
Unfavorable discharge ^d^	RARH vs. LRH	1.26 (0.95, 1.66)	0.109
Hospital cost (per thousand dollars) ^e^	RARH vs. LRH	28.98 (21.02, 36.94)	**< 0.001**

*Abbreviations* RARH, robot-assisted right hemicolectomy; LRH, laparoscopic right hemicolectomy; CCI, Charlson Comorbidity Index; OR, odds ratio; aOR, adjusted odds ratio; aBeta, adjusted Beta; CI, confidence interval; LOS, length of stay

^a^ Adjusted for sex, insurance status, smoking, emergent admission, weekend admission, Hospital bed size, hospital location/teaching status and CCI.

^b^ Adjusted for age group, household income, smoking, emergent admission, weekend admission, admission year, hospital location/teaching status and CCI

^c^ Adjusted for age group, household income, smoking, emergent admission, weekend admission, admission year, Hospital bed size and hospital location/teaching status

^d^ Adjusted for age group, sex, smoking, emergent admission, weekend admission, admission year and CCI

^e^ Adjusted for age group, sex, household income, emergent admission, weekend admission, admission year, hospital location/teaching status and CCI.

^f^ Excluded patients with in-hospital mortality

P-values < 0.05 are shown in bold

## Discussion

Results of the present study revealed that, in octogenarians and nonagenarians with CRC in the US, patients who underwent curative RARH had an approximately 0.24-day significantly shorter hospital LOS than those receiving LRH. This result is independent of other clinical and hospital-related factors including patients’ frailty status. However, RARH was associated with higher total hospital costs than LRH. Nevertheless, the main short-term outcomes—in-hospital mortality, unfavorable discharge, and the risk of perioperative complications—were similar between the two procedures. Stratified analyses by frailty status reveal consistent findings, indicating that the benefit of a shorter LOS with RARH compared to LRH also applies to frail patients. Overall, these findings suggest that RARH is safe and feasible and may offer certain advantages over LRH for this patient subgroup.

Surgical resection is the mainstay of treatment for CRC, making the quality of surgery a significant factor. Because larger randomized controlled trials conducted since the early 2000s consistently demonstrated the non-inferior or equal status of minimally invasive surgeries compared to conventional open resections, open surgery is now seldom performed and laparoscopic colon resection has been established as the standard approach [20–22].

In minimally invasive procedures, specifically robot-assisted colorectal surgery has shown that short-term (operative times, conversion to laparoscopy or laparotomy, intraoperative blood loss/transfusion, intraoperative complications and postoperative morbidity/mortality) and oncological outcomes (excised lymph nodes, resection margins, overall survival (OS) and disease-free survival (DFS) ) have been found equivalent to laparoscopic procedures in older patients (aged 70 years or above) older undergoing elective surgery for CRC [13]. In that study, although LOS was slightly shorter for the robotic group, operative durations in the robotic group significantly surpassed pure laparoscopic procedures. The authors attributed this to the time needed for robotic docking and setup, which might notably impact outcomes due to extended anesthesia and pneumoperitoneum times. In the present study, the hospital LOS was also slightly shorter in the RARH group than that of the LRH group, but we were not able to assess the operation time due to lack of data in the NIS dataset. The computer interface employed in robotic surgery enhances the surgeon’s depth perception, dexterity, and precision of movement, resulting in intuitive actions akin to those in open surgery [23]. Consequently, surgeons well-versed in open procedures are likely to transition more seamlessly to robotic surgery. This improved dynamic of robot-assisted surgery is anticipated to contribute to shorter recovery times and, consequently, result in the further reduction in LOS as compared to the conventional laparoscopic approach.

The present study observed no notable distinctions regarding in-hospital mortality, unfavorable discharge, and overall perioperative complications in the oldest patients undergoing RARH compared to LRH. This observation aligns well with previous research findings [13, 24], implying that ages exceeding 80 or even 90 years should not be regarded as barriers to considering robotic surgery for right-side colon cancer. However, in addition to age itself, some previous reports also emphasized that the presence of frailty accompanied with aging significantly increased the risk of postoperative complications [25–27]. There is a growing recognition that frailty, in addition to chronological age alone, as a more significant predictor of various surgical outcomes in older adults. Frailty encompasses multiple dimensions of health, including physical, cognitive, and nutritional status, which collectively impact a patient’s resilience to surgical stress. For instance, a systematic review and meta-analysis by Marano et al. (2022) highlighted that frailty is a critical predictor of postoperative complications and mortality in older adults undergoing cancer surgery​ [28]. Similarly, Boccardi et al. (2024) emphasized the importance of assessing frailty in geriatric patients to better predict surgical outcomes and tailor perioperative care accordingly​ [29]. This led us to include frailty assessment as one of the critical covariates in the present analysis in addition to the focus on chronological age.

Of note, the present analysis did not evaluated outcomes after discharge; nevertheless, it must also be noted that some previous studies still find equivalent outcomes of overall postoperative morbidity and 90-day mortality between robotic versus laparoscopic procedures in older adult patients [30, 31]. We recommended it to be evaluated in the future prospective studies.

While the higher cost of RARH is discussed, it could be valuable to consider cost-effectiveness in the context of potential benefits related to reduced long-term complications. That said, although robotic surgery is not a cost-saving procedure compared to conventional laparoscopic procedures, the slightly lower hospital LOS observed in our study and others may be one of the few cost-cutting measures associated with robotic surgery. In a prior investigation on the costs of conversion in robotic and laparoscopic surgery, although total hospital costs were greater for the robotic procedure versus laparoscopic, but the occurrence of conversion to open cases decreased the cost differences significantly [32]. The potential reduction in subsequent healthcare utilization may enhance the cost-effectiveness of RARH over time, especially in high-risk elderly populations. This aspect warrants further investigation in future longitudinal studies.

### Strength and limitations

While this study drew from a substantial and reliable national dataset, it’s important to acknowledge a few limitations. First, its retrospective, observational design may limit generalization of results to other populations and not allow inferences of causation or ruling out selection bias. Second, the reliance on data derived from the ICD coding system introduces the potential for coding errors and misclassifications, which can impact the accuracy of the identified diagnoses and procedures. Additionally, important intraoperative parameters, including operative time, laboratory data, and surgeon experience, are not available in the NIS database, precluding their evaluation in this study. Although previous research has focused on the possible impact of ileocolic anastomosis technique on outcomes in minimally invasive right colectomies [33], this could not be taken into account in the current analysis because of a lack of data. Lastly, because NIS data are recorded only to patient discharge and long-term follow-up data are not available, patients’ long-term oncological outcomes could not be assessed.

## Conclusion

LRH and RARH performed for right-sided colon cancer in octogenarians and nonagenarians have similar short-term outcomes, including in-hospital mortality, perioperative complications and unfavorable discharge. RARH is independently associated with a slightly shorter hospital LOS but still has greater total hospital costs than LRH. These findings also apply to frail patients. Overall, our analyses suggest these minimally invasive surgery types are both viable for the oldest old population with right-side colon cancer, and RARH may offer certain advantages over LRH.

## Electronic supplementary material

Below is the link to the electronic supplementary material.

Supplementary Material 1

## Data Availability

No datasets were generated or analysed during the current study.
