# Supplementary material for: Robot-assisted vs. laparoscopic right hemicolectomy in octogenarians and nonagenarians: an analysis of the US nationwide inpatient sample 2005–2018
Source: Aging Clin Exp Res. 2024 Sep 23;36(1):193. doi: 10.1007/s40520-024-02833-4 (PMC11420325; doi:10.1007/s40520-024-02833-4)
Supplement: Supplementary file 1 — Supplementary Material 1 [file 40520_2024_2833_MOESM1_ESM.docx]

**Supplemental Table S1. ICD codes used in the study.**

|  | **ICD-9 code** | **ICD-10 code** |
| --- | --- | --- |
| Right-side colon cancer | **CM**: 153.0, 153.1, 153.4, 153.6 | **CM**: C18.0, C18.2, C18.3, C18.4 |
| Laparoscopic right  hemicolectomy | **PCS:** (17.33 or 45.73) and 17.4 | **PCS:** (0DTF8ZZ, 0DTF4ZZ or 0DTF7ZZ or 0DTF8ZZ) and (8E0W3C or 8E0W4C or 8E0W7C or 8E0W8C or 8E0WXC) |
| Robot-assisted right  hemicolectomy | **PCS:** 17.33, 45.73 and 54.21 | **PCS:** 0DTF4ZZ, 0DTF7ZZ, 0DTF8ZZ, (0DTF4ZZ or 0DTF7ZZ or 0DTF8ZZ) and (8E0W3C or 8E0W4C or 8E0W7C or 8E0W8C or 8E0WXC) |
| Metastatic disease | **CM**: 197, 198, 199 | **CM**: C78, C79, C80 |
| Acute myocardial infarction | **CM**: 410  DXCCS: 100 | **CM**: I21  DXCCSR_CIR009 >0 |
| Venous thromboembolism | **CM**: 415, 451-453, 671, 673, 997.2 | **CM**: I260, I269, I801-803, I808, I809, I820-I823, I828, I829, O082, O223, O871, O882, I81, I82 |
| Pneumonia | **CM**: 115.05, 115.15, 115.95, 480, 481, 482.3, 482.8, 484, 486 | **CM**: A48.1, J12 - J18, B39.2, B39.5, B39.9, A22.1, B25.0, A37.91, B44.0 |
| Sepsis | **CM**: 995.9, 996.64, 038, 999.3, 790.7, 041, 785.52 | **CM**: R78.81, A41, R65.2, T81.4, T80.2, A42.7, A22.7, B37.7, A26.7, A28.2, A54.86, B00.7, A32.7, A24.1, A39.2, A20.7, A21.7, A48.3 |
| Infection | **CM**: 001-041; 045-139, 599.0 | **CM**: L00-L08, B99, T81.43, O86.03, N39.0 |
| Major blood loss | **CM**: 459.0, 285.1, 998.1  **PCS:**39.98, 99.0 | **CM**: R58, D62  **PCS:** 0W380ZZ, 0W383ZZ, 0W384ZZ, 302* |
| Respiratory failure/  Mechanical ventilation | **CM**: 518.5, 518.81-518.84  **PCS**: 96.72, 93.90, 96.01-96.05 | **CM**: J80, J81.0, J95.2-J95.8, J96.00, J96.90  **PCS**: 5A1935Z, 5A1945Z, 5A1955Z, 5A09357, 5A09457, 5A09557, 09HN7BZ, 09HN8BZ, 0CHY7BZ 0CHY8BZ, 0DH57BZ, 0DH58BZ 0BH17EZ, 0BH18EZ, 0B717DZ 0B718DZ, 0BH07DZ |
| Acute kidney injury | **CM**: 584  DXCCS: 157 | **CM**: N17  DXCCSR_GEN002 >0 |
| Postoperative ileus | **CM**: 560 | **CM**: K91.3 |
| Abdominal abscess/ fistula | **CM**:998.59, 998.6 | **CM**: K68.11, T81.83 |
| Wound disruption | **CM**: 998.31, 998.32 | **CM**: T81.3 |
| Smoking | **CM**: 305.1, V15.82, 989.84 | **CM**: Z71.6, Z72.0, Z86.43, Z87.891, F17, O99.33, T65.2 |
| Chronic kidney disease | **CM**: 403.01, 403.11, 403.91, 404.02, 404.03, 404.12, 404.13, 404.92, 404.93, 582, 583.0-583.7, 585, 586, 588.0, V42.0, V45.1, V56 | **CM**: I12.0, I13.1, N03.2-N03.7, N05.2-N05.7, N18, N19, N25.0, Z49.0-Z49.2, Z94.0, Z99.2 |
| Ischemic heart disease | **CM**: 410–414 | **CM**: I25 |
| Congestive heart failure | CM_CHF=1 | **CM**: I09.9, I11.0, I13.0, I13.2, I25.5, I42.0, I42.5-I42.9, I43, I50, P29.0 |
| Chronic obstructive  pulmonary disease | **CM**: 491, 492, 496  CM_CHRNLUNG=1 | **CM**: J40-44 |
| Cerebrovascular disease | **CM**: 362.34, 430-438 | **CM**: G45, G46, H34.0, I60-I69 |
| Severe liver disease | **CM**: 456.0–456.2, 572.2-572.8 | **CM**: I85.0, I85.9, I86.4, I98.2, K70.4, K71.1, K72.1, K72.9, K76.5, K76.6, K76.7 |
| Systemic connective  tissue disorders | **CM**: 446.5, 710, 714.0-714.2, 714.8, 725  CM_ARTH=1 | **CM**: M05, M06, M31.5, M30-M36 |
| Coagulopathy | CM_COAG=1 | **CM**: D65-69 |
| Diabetes | CM_DM=1, CM_DMCX=1 | **CM**: E10-E14 |
| Obesity | **CM**: 278.0, 278.1, V85.21-V85.45  CM_OBESE=1 | **CM**: E66, Z68.25-Z68.45 |

Abbreviation: ICD, International Classification of Diseases; CM, Clinical Modification; PCS, Procedure Coding System.
